# Experimental West Nile Virus Infection in Rabbits: An Alternative Model for Studying Induction of Disease and Virus Control

**DOI:** 10.3390/pathogens4030529

**Published:** 2015-07-14

**Authors:** Willy W. Suen, Muhammad J. Uddin, Wenqi Wang, Vienna Brown, Danielle R. Adney, Nicole Broad, Natalie A. Prow, Richard A. Bowen, Roy A. Hall, Helle Bielefeldt-Ohmann

**Affiliations:** 1 School of Veterinary Science, University of Queensland, Gatton, QLD 4343, Australia; E-Mails: w.suen@uq.edu.au (W.W.S.); m.uddin2@uq.edu.au (M.J.U.); w.wang3@uq.edu.au (W.W.); 2 Department of Biomedical Sciences, Colorado State University, Fort Collins, CO 80523, USA; E-Mails: vienna.brown@colostate.edu (V.B.); rbowen@rams.colostate.edu (R.A.B.); 3 Department of Microbiology, Immunology and Pathology, Colorado State University, Fort Collins, CO 80523, USA; E-Mail: danielle.adney@colostate.edu; 4 QIMR Berghofer Medical Research Institute, Brisbane, QLD 4006, Australia; E-Mails: nicole.broad@qimrberghofer.edu.au (N.B.); Natalie.Prow@qimrberghofer.edu.au (N.A.P.); 5 Australian Infectious Diseases Research Centre, University of Queensland, St Lucia, QLD 4072, Australia; E-Mail: roy.hall@uq.edu.au; 6 School of Chemistry and Molecular Bioscience, University of Queensland, St Lucia, QLD 4072, Australia

**Keywords:** West Nile virus, animal models, pathogenesis, rabbit

## Abstract

The economic impact of non-lethal human and equine West Nile virus (WNV) disease is substantial, since it is the most common presentation of the infection. Experimental infection with virulent WNV strains in the mouse and hamster models frequently results in severe neural infection and moderate to high mortality, both of which are not representative features of most human and equine infections. We have established a rabbit model for investigating pathogenesis and immune response of non-lethal WNV infection. Two species of rabbits, New Zealand White (*Oryctolagus cuniculus*) and North American cottontail (*Sylvilagus* sp.), were experimentally infected with virulent WNV and Murray Valley encephalitis virus strains. Infected rabbits exhibited a consistently resistant phenotype, with evidence of low viremia, minimal-absent neural infection, mild-moderate neuropathology, and the lack of mortality, even though productive virus replication occurred in the draining lymph node. The kinetics of anti-WNV neutralizing antibody response was comparable to that commonly seen in infected horses and humans. This may be explained by the early IFNα/β and/or γ response evident in the draining popliteal lymph node. Given this similarity to the human and equine disease, immunocompetent rabbits are, therefore, a valuable animal model for investigating various aspects of non-lethal WNV infections.

## 1. Introduction

West Nile virus (WNV) is an important re-emerging neurotropic arbovirus, that continues to cause severe outbreaks world-wide. However, the majority of WNV infections in humans and horses, the incidental hosts of the transmission cycle, is non-lethal and subclinical [1,2,3,4,5]. The reported mortality rate in humans and horses from neuroinvasive WNV disease is <1% and ~10%, respectively, of those infected [1,2,3,4,5]. But the economic burden of non-lethal WNV disease is substantial. Nolan *et al.* has reported an estimated cost of $42 million for non-lethal West Nile fever (WNF) human cases in Texas alone during the period spanning 2002 to 2011 [6]. An estimated further $70 million was reported for West Nile neuroinvasive disease (WNND) cases in the same period [6]. Given the low case-fatality rate of ~9% of WNND in humans, the majority of the $70 million would have been attributed to non-lethal neurological WNV cases [3]. This highlights the significance in investigating the mechanisms of non-lethal WNV disease.

Currently, the mouse and hamster are the main small animal models for investigating WNV-associated pathogenesis and host immune response in humans and horses [7,8,9]. While these rodent models typically produce severe encephalitis after virulent WNV infection, the level of central nervous system (CNS) infection in these small animal models often is too severe to be reflective of most human and equine disease [7,8]. Minimal levels of virus replication take place in the CNS of most human and equine infection, unless the individuals are immunocompromized [10,11,12,13]. This important difference suggests that the neuropathogenesis of WNV in immunocompetent rodents poorly reflects what happens in immunocompetent humans and horses. In addition, the relatively higher susceptibility of the mouse model means that investigations into mechanisms of virus control will often require the use of attenuated WNV strains [14,15]. But whether these mechanisms reflect how most healthy humans and horses naturally overcome virulent WNV infection is questionable.

Given these limitations associated with the current rodent models, there is a need to test and establish alternative small animal infection models to study natural mechanisms of virus control and induction of WNV disease. While non-human primate (NHP) and direct horse infection models can be used to better understand WNV pathogenesis, the cost and logistics are greatly limiting.

The present study established an alternative small animal model in laboratory New Zealand White rabbits (NZWR; *Oryctolagus cuniculus*) for investigating WNV-induced disease and host immune response. Comparative experimental infection was conducted in wild-caught North American cottontail rabbits (CTR; *Sylvilagus* sp.). A contemporary Australian equine-pathogenic outbreak strain, WNV_NSW2011_, was used as the main strain of interest as it has been shown to be highly virulent in the weanling CD1 Swiss mouse model and of intermediate virulence in the young adult model [16]. A 2012 North American WNV isolate (WNV_TX8667_) and the Murray Valley encephalitis virus (MVEV) prototype strain, MVE_1-51,_ were used as virulent flavivirus controls.

The resultant mild clinical course without mortality, low viremia, minimal to absent CNS infection, mild to moderate neuropathology, and fast neutralizing antibody response in WNV-infected rabbits mimic closely the features of most human and equine WNV infections. Based on the virus kinetics, the robust type I and/or II interferon (IFN-I and -II) responses detected in the draining popliteal lymph node (PLN) and brain appeared to be important as first defence against early virus replication. Neutralizing antibodies then additionally restricted and resolved WNV infection. This consistently resistant phenotype in rabbits against virulent WNV infection suggests that rabbits are a superior model for studying resolution of virulent WNV infections compared to the relatively more susceptible mouse model. The rabbit model presented here will be valuable for identifying novel prognostic indicators, and for developing new therapeutics and prevention strategies. The model will also be amenable for studies of the effect of immunosuppression or co-infection on the outcome of WNV-infection, which are well-known risk factors for development of severe neurological disease in humans and horses [11,17].

## 2. Results

### 2.1. Subclinical and Non-Lethal Infection

A total of 63 rabbits were experimentally infected with outbreak strains of flavivirus originally isolated from Australia (WNV_NSW2011_ and MVE_1-51_ [16,18]) and North America (WNV _TX8667_). Inocula were administered intradermally in the left hind footpad at the dose of 10^5^ TCID_50_ for NZWRs infected with WNV_NSW2011_ and MVE_1-51,_ or 10^5^ PFU for CTRs infected with WNV_NSW2011_ and WNV_TX8667_. Table 1 summarizes the assignment of rabbits into each challenge group. None of the infected rabbits succumbed to the virus challenge. The only clinical sign of illness was a mild to moderate fever evident on day 1 post-infection (pi) in weanling NZWRs (Figure S1A; WNV_NSW2011_: mean: 39.71 °C ± 0.069, *n* = 24; MVE_1-51_: mean: 39.92 °C ± 0.13, *n* = 21; Control: mean: 39.17 °C ± 0.076, *n* = 6). No febrile response was detected in any of the infected adult NZWRs and CTRs (Figure S1B). Enlargement of the draining and contralateral PLNs was detected on palpation during clinical examination of the NZWRs (Figure S1C–F). However, there were no obvious signs of neurological deficit or dysfunction, nor were there any significant changes in weight of the infected rabbits.

**Table 1:** Assignment and number of rabbits per challenge group

Rabbit Species	New Zealand White Rabbit (NZWR)					Cottontail Rabbit (CTR)		
Age	Weanlings (4–5 Weeks Old, ~500–700 g)			Adults (>3 Months Old, ~3000 g)		Mixed Age (~250–900 g)		
Inoculum	WNV_NSW2011_	MVE_1-51_	Sham	WNV_NSW2011_	MVE_1-51_	WNV_NSW2011_	WNV_TX8667_	Sham
Termination day (pi)	Group 1	Group 2	Control	Group 3	Group 4	Group 5	Group 6	Control
3	3	3	N/D	N/D	N/D	3	3	1
7	9	6	3	3	3	3	3	1
12	6	6	N/D	N/D	N/D	N/D	N/D	N/D
18	6	6	3	N/D	N/D	N/D	N/D	N/D
Totals	24	21	6	3	3	6	6	2

N/D: not done.

### 2.2. Neuropathology

**Figure 1:**
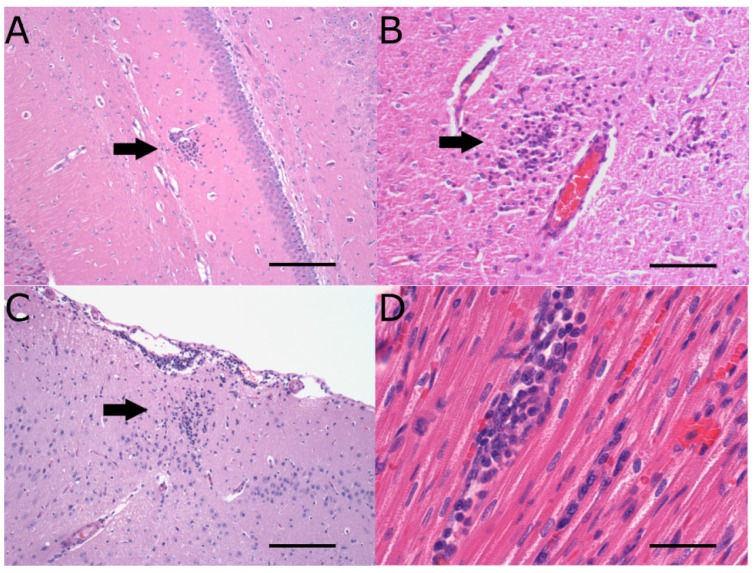
Histopathology of the central nervous system (CNS) and heart. Mononuclear leukocytic infiltrate (black arrow) in the neuropil of brains from NZWRs infected with WNV_NSW2011_ (**A**), MVE_1-51_ (**B**) and CTRs- infected with WNV_TX8667_ (**C**) on day 7 pi. Mononuclear leukocytic infiltrate in myocardium of a NZWR infected with WNV_NSW2011_ on day 7 pi (**D**). Panels (**A**–**D**) were stained with H&E stain. Magnifications: (**A**) 10×; (**B**) 20×, (**C**) 10×, and (**D**) 40×. Scale bar in each panel represents 150 µm (**A**,**C**), 100 µm (**B**), and 30 μm (D).

The neuropathology observed in rabbits infected with WNV_NSW2011_, WNV_TX8667,_ and MVE_1-51_ consisted of mild to moderate mononuclear leukocytic, and occasionally heterophilic, infiltration in the CNS, with the latter two strains producing the most frequent and severe lesions (Figure 1A–C). There was a trend for weanling NZWRs to develop more severe neuropathology than adults (Table 2). Table 3 summarizes the kinetics of neuropathology observed in the different virus challenge groups at days 3, 7, 12, and 18 pi. Most severe and extensive leukocyte infiltration in the CNS was observed on day 7 pi for both weanling NZWR groups (Table 3). However, moderate neuropathology was still evident on day 12 pi in brains of weanling NZWRs infected with MVE_1-51_. The incidence of MVE_1-51_-induced neuropathology in weanling NZWRs on day 7 was significantly higher than those of the sham-inoculation control group (*p* = 0.0022), of adult NZWRs infected with MVE_1-51_ (*p* = 0.0333) and of weanling NZWRs infected with WNV_NSW2011_ (*p* = 0.0108). Mild to moderate perivascular cuffing, extensive gliosis, and occasional neuronal degeneration with neuronophagia were evident in brains of weanling NZWRs infected with MVE_1-51_ at all time-points beyond day 7 pi. This contrasts with the relatively milder lesions seen in weanling NZWRs infected with WNV_NSW2011_ (Figure 1A). Neuropathology seen in the WNV_NSW2011_-infected CTRs was comparable to that in weanling NZWRs infected with the same strain (Table 3). CTRs infected with WNV_TX8667_ showed more extensive and severe lesions in the CNS than ones infected with WNV_NSW2011_, though there was no evidence of neuronal degeneration in the former group (Figure 1C).

**Table 2:** Comparison of the peak neuropathology scores in NZWRs on day 7 pi.

Neuropathology	Virus strain	Weanling	Adults
Meningitis (Brain)	WNV_NSW2011_	2	1
	MVE_1-51_	2	1
Encephalitis (Brain)	WNV_NSW2011_	2	1
	MVE_1-51_	3	1
Meningitis (Olfactory bulbs)	WNV_NSW2011_	2	1
	MVE_1-51_	2	1
Encephalitis (Olfactory bulbs)	WNV_NSW2011_	2	0
	MVE_1-51_	2	0
Spinal Meningitis	WNV_NSW2011_	2	0
	MVE_1-51_	1	0
Myelitis	WNV_NSW2011_	2	0
	MVE_1-51_	2	0

Neuropathology was scored using a 5-tiered system, with 0 indicating normal and 5 indicating severe and diffuse lesions. This scoring system takes into account of the severity and extensiveness of the lesions (see Table 4).

**Table 3:** Comparison of neuropathology kinetics.

	Day 3 pi	Day 7 pi	Day 12 pi	Day 18 pi
WNV_NSW2011_ (weanling NZWRs)	Mn and rare htl near CVO and neuropil of brain (peak score = 1, *n* = 2/3)	Mn in meninges, neuropil of brain, olfactory bulbs, spinal cord, perineurium and/or nerve fibres of spinal nerves in cauda equine (peak score = 2, *n* = 4/9)	Mn in meninges, neuropil of brain and cranial cervical spinal cord (peak score = 1, *n* = 4/6)	Mn in meninges, neuropil of brain near CVO and cranial cervical spinal cord (peak score = 1, *n* = 2/6)
MVE_1-51_ (weanling NZWRs)	No pathology (*n* = 0/3)	Mn with occasional htl in meninges, neuropil of brain (extensive with neuronophagia), olfactory bulbs, spinal cord, perineurium of spinal nerves and/or dorsal root ganglion in cauda equine (peak score = 3, *n* = 6/6)	Mn,with occasional htl in meninges, neuropil of brain (extensive) with neuronophagia^+^ (peak score = 3, *n* = 4/6)	Mn,with occasional htl in meninges, neuropil of brain (extensive)^+^ (peak score = 2, *n* = 2/6)
WNV_NSW2011_ (CTRs)	No pathology (*n* = 0/3)	Mn in meninges, neuropil of brain and spinal cord (peak score = 2, *n* = 1/3)	N/D	N/D
WNV_TX8667_ (CTRs)	No pathology (*n* = 0/3)	Mn in meninges and neuropil of brain (extensive; peak score = 3, n 2/3)	N/D	N/D

Mn, mononuclear leukocytes; Htl, heterophils; CVO, circumventricular organ; peak score, peak neuropathology score in the CNS (out of 5). ^+^ Spinal cord and olfactory bulbs were not examined, N/D: not done.

In addition to the CNS, histopathology of the peripheral nervous system was analyzed in a subset of animals. There was no frank lesion in the sciatic nerve ipsilateral to the footpad inoculation, except for one CTR infected with WNV_NSW2011_ and sacrificed on day 3 pi. However, mild to moderate peri- and/or epineuritis was commonly observed in the ipsilateral nerve on day 3 pi and the contralateral nerve on day 3 and 7 pi (Table S3).

**Figure 2:**
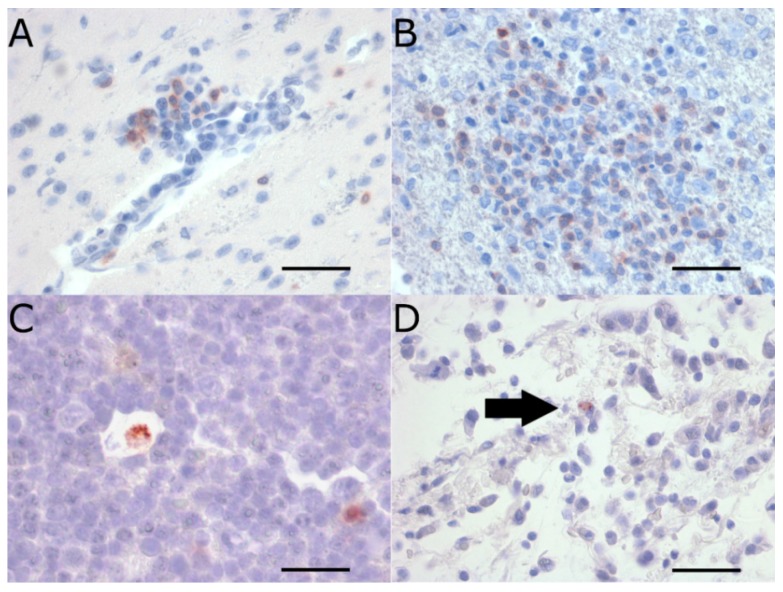
CD3+ T cells in the brain (**A**,**B**), and viral NS1 antigen in the draining popliteal lymph node (PLN) (**C**) and dermis (**D**). CD3+ T lymphocytes (red stain) infiltrating from cerebral blood vessel (**A**) in a NZWR infected with WNV_NSW2011_ on day 7 pi, and in neuropil of brain of a NZWR infected with MVE_1-51_ (**B**); Flavivirus NS1 antigen positive leukocyte (red strain) in the paracortical zone of the draining PLN (**C**); Arrow in panel (**D**) indicates viral NS1 staining in an individual leukocyte in the deep interstitium of the footpad dermis, ipsilateral to inoculation (**D**); Panels (**A**–**D**) were stained with AEC substrate and counterstained with Meyer’s hematoxylin. Magnifications: (**A**) 40×, (**B**) 40×, (**C**) 40× cropped, and (**D**) 40×. Scale bar in each panel represents 30 μm (**A**,**B**,**D**) and 20 μm (**C**).

Immunolabeling for leukocyte markers established that approximately half of the CNS-infiltrating leukocytes for rabbits infected with WNV_NSW2011_ and MVE_1-51_ were CD3 positive T lymphocytes (Figure 2A,B). No signal was detected when sections were labeled for the myelomonocytic marker MAC387. However, this immune-marker appeared to only stain a subset of the monocytic/histiocytic cell lineage in rabbits, as evident when applied on spleen sections. There was no histological evidence of mature plasma cells in any of the CNS sections examined. Due to the lack of rabbit-specific or cross-reactive antibodies, the remaining population of infiltrating mononuclear leukocytes could not be phenotypically identified at this point in time.

### 2.3. Extraneural Pathology

Lymphocyte activation, as suggested by formation of secondary follicles, paracortical hyperplasia, hypercellularity of the medulla, and hypertrophy of post-capillary endothelial venules, was observed commonly in the draining PLN on day 3 and 7 pi for rabbits infected with the three strains of flaviviruses (Table S1). Activation of the contralateral PLN was also occasionally observed in weanling NZWRs (WNV_NSW2011_: *n* = 1/2 examined on day 7 pi; MVE_1-51_: *n* = 1/3 examined on day 7 pi), but not in adult rabbits on day 7 pi. Notably, all examined contralateral PLNs of CTRs infected with either strain of WNV were activated to varying degree (WNV_NSW2011_ and WNV_TX8667_: *n* = 2/2 on day 3 pi, *n* = 3/3 on day 7 pi). Where present, there was mild to moderate activation of most tracheobronchial lymph nodes examined, regardless of virus challenge groups. The bronchus-associated lymphoid tissues were also observed to be mildly activated in WNV_NSW2011_ infected weanling NZWRs on day 7 pi (*n* = 3/3).

Major organs were also examined for histopathological lesions in a subset of rabbits from each virus challenge group. Mild focal to multifocal lymphohistiocytic myocarditis with or without epi- and endo-carditis was consistently observed in rabbits infected with WNV_NSW2011_ on day 7 pi (Figure 1D, *n* = 6/6 weanling NZWRs, n = 1/3 adult NZWRs and *n* = 3/3 CTRs) and occasionally in animals infected with MVE_1-51_ (*n* = 1/3 weanling and *n* = 1/3 adult NZWRs) and WNV_TX8667_ (*n* = 1/3 of CTRs). All other organs examined (lungs, kidneys, liver, adrenals, thymus, and eyes) were histologically unremarkable.

### 2.4. Viral Burden in the CNS

Despite the induction of neuropathology, no infectious virus, viral protein, or viral RNA was detected in the brains of NZWRs and CTRs infected with WNV_NSW2011_. Similar absence of infectious virus was noted in the brains of CTRs infected with WNV_TX8667_. Only trace levels of MVE_1-51_ viral RNA were detected in 4/7 of brains that had an encephalitis score of 2 or more (*n* = 4/12 tested, median: 22-fold [increase in viral burden over control], interquartile range [IQR]: 5–43). This suggests a potential correlation between the degree of brain viral load and the degree of neuropathology. Amongst the MVE_1-51_ positive brains, two were harvested on day 7 pi, one on day 12 pi, and one on day 18 pi. However, virus isolation and/or immunohistochemistry failed to detect infectious virus or viral protein from these viral RNA positive brains.

### 2.5. Peripheral Virus Replication

**Figure 3:**
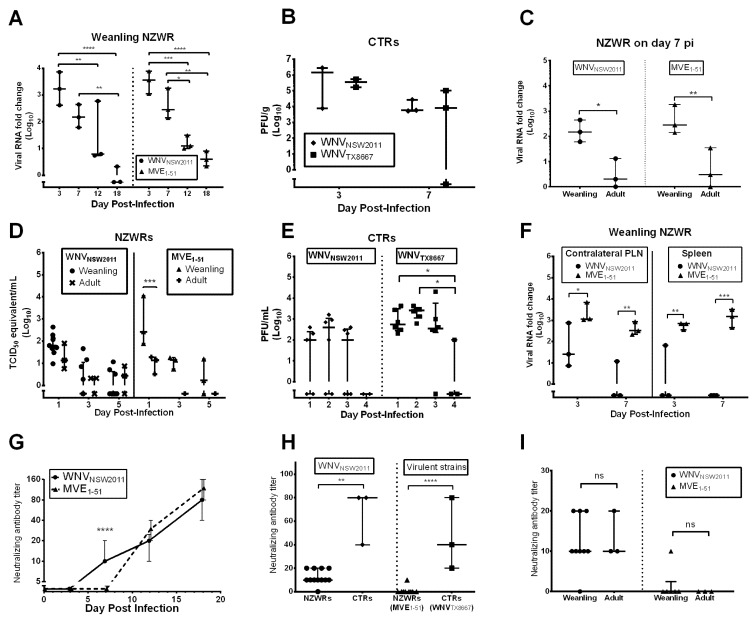
Peripheral virus dissemination and neutralizing antibody response. Viral load in the draining PLN of weanling and adult NZWRs (**A**,**C**) and CTRs (**B**). Viremia in NZWRs (**D**) and CTRs (**E**). Viral burden in the contralateral PLN and spleen (**F**). Viral RNA in NZWRs was quantified using qRT-PCR, while virus load in CTRs was quantified by plaque assay. Neutralizing antibody kinetics in weanling NZWRs (**E**), comparison of neutralizing antibody titer between NZWRs and CTRs on day 7 pi (**F**), and between weanling and adult NZWRs on day 7 pi (**I**). Note for (**F**), the NZWR groups include data from both weanling and adult groups to give a closer comparison to the CTR groups. PRNT_90_ was used to determine neutralizing antibody titers. Each data point for panels (**A**–**F**,**H**,**I**) represents titer for one animal. Horizontal line in these panels indicates the median titer of each time-point. For (**E**), the points on the line indicate the median titer of each time point (*n* = 3 for day 3 pi, 6–9 for day 7 pi and 6 for both day 12 and 18 pi). Error bars indicate the interquartile range or range. Two-way ANOVA with post-test Sidak was performed on the log_10_(Y+1) transformed means of titers in each time-point for panels (**A**–**G**,**I**). One-way ANOVA with post-test Tukey was performed on the log_10_(Y+1) transformed means of titers in each challenge group for panel (**H**). Statistical significance thresholds: ^ns^ not significant, * *p* ≤ 0.05, ** *p* ≤ 0.01, *** *p* ≤ 0.001, **** *p* ≤ 0.0001.

The main site of peripheral replication of WNV_NSW2011_ and MVE_1-51_ in weanling NZWRs appeared to be the draining PLN. From day 3 to 12 pi, viral RNA was detected in this lymph node, with peak levels reached on day 3 pi (Figure 3A; WNV_NSW2011_: median: 1678-fold, range: 414–7194; MVE_1-51_: median: 3666-fold, range: 1121–7750). This peak was significantly higher than levels detected on day 12 (WNV_NSW2011_: *p* = 0.0082; MVE_1-51_: *p* = 0.0008) and 18 pi (WNV_NSW2011_: *p* < 0.0001; MVE_1-51_: *p* < 0.0001). For the MVE_1-51_ group, the day 7 pi viral burden was also significantly higher than on day 12 (*p* = 0.0433) and 18 pi (*p* = 0.0041). By day 18 pi, viral RNA level was mostly below the detection limit. The overall viral burden in this lymph node was not significantly different between these two virus challenge groups (F_1,16_ = 0.8479, *p* = 0.3708). On day 7 pi, the viral burden in the draining PLN of weanling NZWRs was significantly higher than that of adult NZWRs, regardless of flavivirus strain inoculated (Figure 3C, WNV_NSW2011_: *p* = 0.0141; MVE_1-51_: *p* = 0.0065).

Similar kinetics of virus replication in the draining PLN was observed in CTRs infected with the two strains of WNV (Figure 3B). A peak median viral load of 1.4 × 10^6^ (range: 7.6 × 10^3^−2.8 × 10^6^) and 3.5 × 10^5^ PFU/g (range: 1.6 × 10^5^–5.3 × 10^5^) was reached on day 3 pi for CTRs infected with WNV_NSW2011_ and WNV_TX8667_, respectively (Figure 3B). But the viral burden was not significantly different between day 3 and 7 pi (F_1,5_ = 3.060, *p* = 0.1406) and between the two virus challenge groups (F_1,5_ = 0.3991, *p* = 0.5553). Viral RNA assay showed that the amount of WNV_NSW2011_ RNA in the draining PLN of WNV_NSW2011_-infected CTRs was 0.4 and 1.8-fold of that in weanling NZWRs infected with the same virus on day 3 and 7 pi, respectively. This suggests comparable viral burden in the draining PLN between the two species.

Flavivirus NS1 antigen was detected by IHC in pleomorphic leukocytes, suggestive of macrophages and/or dendritic cells, in the paracortical zone of draining PLNs (Figure 2C). Lymph nodes from day 3 pi were more consistently IHC positive for NS1 antigen than on day 7 pi (Table S1). IHC of the footpad, ipsilateral to the inoculation, also revealed NS1 antigen in leukocytes in multiple sites of the deep dermis, in one MVE_1-51_ infected weanling NZWR culled on day 3 pi (Figure 2D). These dermal leukocytes were morphologically consistent with macrophages or dendritic cells.

### 2.6. Peripheral Virus Dissemination

Despite evidence of virus replication in the draining PLN, the associated viremia was transient and of low magnitude for all virus challenge groups (Figure 3D,E). Peak viremia was reached on day 1 pi for weanling (median: 64 TCID_50_ equivalent/mL, IQR: 40–162) and adult NZWRs (median: 14 TCID_50_ equivalent/mL, range: 6–80) infected with WNV_NSW2011_ (Figure 3D). MVE_1-51_ infection in weanling NZWRs resulted in a higher median viremia peak at 270 TCID_50_ equivalent/mL (range: 78–11234) on day 1 pi, which was significantly higher than the median peak of 14 TCID_50_ equivalent/mL (range: 3–19) reached in the adult group (Figure 3D, *p* = 0.0063). The overall viremia was significantly different between the two weanling NZWR groups (F_1,30_ = 7.377, *p* = 0.0109), however, there was no single time-point where a significant difference was observed.

A comparable kinetics of viremia was seen in the WNV-infected CTRs (Figure 3E). Peak viremia occurred on day 2 pi (WNV_NSW2011_: median: 400 PFU/mL, IQR: 0–1075; WNV_TX8667_: median: 2600 PFU/mL, IQR: 1125–3225). The overall viremia between these two CTR groups was significantly different (F_1.34_ = 11.28, *p* = 0.0019). But as for the comparison between the two weanling NZWR groups, there was no single time-point where the difference was significant. For all challenge groups, viremia dropped below the detection limit by day 4 to 5 pi.

The degree of virus replication in other peripheral tissues was limited in WNV_NSW2011_-infected rabbits. Sites other than the draining PLN where WNV_NSW2011_ RNA was detected in infected weanling NZWRs and CTRs included the contralateral PLN and/or the spleen. Low levels of WNV_NSW2011_ RNA were frequently detected in the contralateral PLN on day 3 pi for NZWR weanlings (*n* = 3/3) and CTRs (*n* = 2/3). But the viral burden in this lymph node was considerably less than that of the draining PLN (Figure 3F, weanling NZWRs: median: 26-fold, range: 7–750; CTRs: median: 1.6-fold, range: 0–116). It was rare to detect viral RNA in this tissue on day 7 pi (weanling NZWR: *n* = 1/3, viral burden: 12-fold; adult NZWR: *n* = 0/3; CTRs: *n* = 0/3). Minimal levels of viral RNA were infrequently detected in the spleen collected from WNV_NSW2011_-infected weanling NZWRs on day 3 pi (*n* = 1/3; viral burden: 67-fold). No viral RNA was detected in spleen samples from the remaining WNV_NSW2011_-challenged groups (day 7 pi weanling and adult NZWRs, day 3 and 7 pi CTRs).

In contrast, when compared to weanling NZWRs infected with WNV_NSW2011_, ones infected with MVE_1-51_ had significantly higher levels of viral RNA in the contralateral PLN (Figure 3F, day 3 pi: median: 1152-fold, range: 1075–7033, *p* = 0.0384; day 7 pi: median: 324-fold, range: 217–867, *p =* 0.0066) and spleen (Figure 3F, day 3 pi, median: 690-fold, range: 362–704, *p* = 0.0037 and day 7 pi: median: 1531, range: 461–3236, *p* = 0.0003). There was also infrequent detectable MVE_1-51_ RNA in the contralateral PLN (*n* = 1/3, 644-fold) and spleen of MVE_1-51_ infected adult NZWRs (*n* = 1/3, 41-fold), showing a higher capacity for MVE_1-51_ to disseminate to other lymphoid tissues in both age groups of NZWRs.

### 2.7. Neutralizing Antibody Response

In order to study the potential host factors restricting viremia, the virus-neutralizing antibody response was measured using a plaque reduction neutralization test (PRNT_90_). A pan-specific flavivirus blocking ELISA was also performed as described [19], with detection of flavivirus specific antibodies in both weanling and adult NZWR MVE_1-51_ challenged groups. An increasing titer of neutralizing antibodies against the inoculated virus was detected, beginning on day 7 and 12 pi for WNV_NSW2011_- and MVE_1-51_- challenged weanling NZWRs, respectively (Figure 3G). The overall neutralizing antibody titers between the two weanling NZWR groups was not significantly different to each other (F_1,8_ = 2.425, *p* = 0.1253); however, the titers reached on day 7 pi for WNV_NSW2011_-challenged weanling NZWRs (median: 10, IQR: 10–20) was significantly higher than those observed in MVE_1-51_ challenged weanling NZWRs (median: 0, IQR: 0–2.5, *p* < 0.0001). The neutralizing antibody titers in the adult NZWR groups on day 7 pi (WNV_NSW2011_: median: 10, IQR: 10–20; MVE_1-51_: median: 0, IQR: 0–0) were comparable to those in the weanlings challenged with the corresponding virus (Figure 3I, F_1,17_ = 0.02651, *p* = 0.8726). Notably, neutralizing antibody titers in WNV_NSW2011_-challenged CTRs were significantly higher than those in WNV_NSW2011_-challenged NZWRs on day 7 pi (Figure 3H; CTR median: 80, IQR: 40–80, *p* = 0.0094). Titers in CTRs infected with WNV_TX8667_ were comparable to those in CTRs infected with WNV_NSW2011_ (*p* = 0.3748). But between the two virulent-virus controls of each rabbit species, CTRs infected with WNV_TX8667_ had significantly higher neutralizing antibody titers than NZWRs infected with MVE_1-51_ on day 7 pi (Figure 3H, *p* < 0.0001).

### 2.8. IFN-I and -II Transcription Levels in the Draining Popliteal Lymph Node and Brain

Given the general resistance of NZWRs and CTRs to WNV and MVEV challenge and the early neutralizing antibody response consequently observed, the type I and II interferon (IFN-I and -II) responses in the brain and draining PLN were investigated.

**Figure 4:**
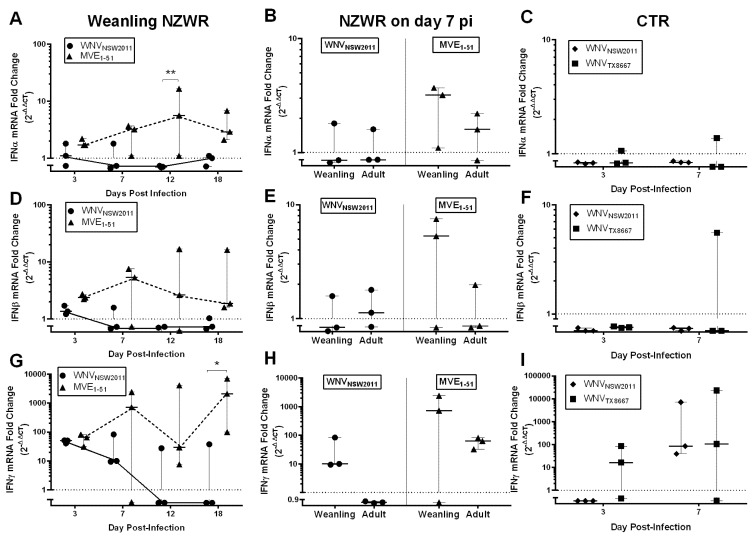
Type I and II IFN transcription kinetics in the brain. IFNα (**A**–**C**), β **(D**–**F)** and γ (**G**–**I**) transcription kinetics in the brain of weanling NZWRs (**A**,**D**,**G**) and CTRs (**C**,**F**,**I**). A comparison of the transcription profile on day 7 pi between weanling and adult NZWRs (**B**,**E**,**H**). Each data point represents the fold-change in transcription in one animal. Horizontal solid line in each time-point represents the median fold-change, with error bars indicating the range. Dotted line at Y = 1 signifies one-fold (basal level of transcription). Two-way ANOVA with post-test Sidak was performed on the log_10_(Y + 1) transformed mean of fold-change in each time-point. Statistical significance thresholds: * *p* ≤ 0.05, ** *p* ≤ 0.01, *** *p* ≤ 0.001, **** *p* ≤ 0.0001.

**Figure 5:**
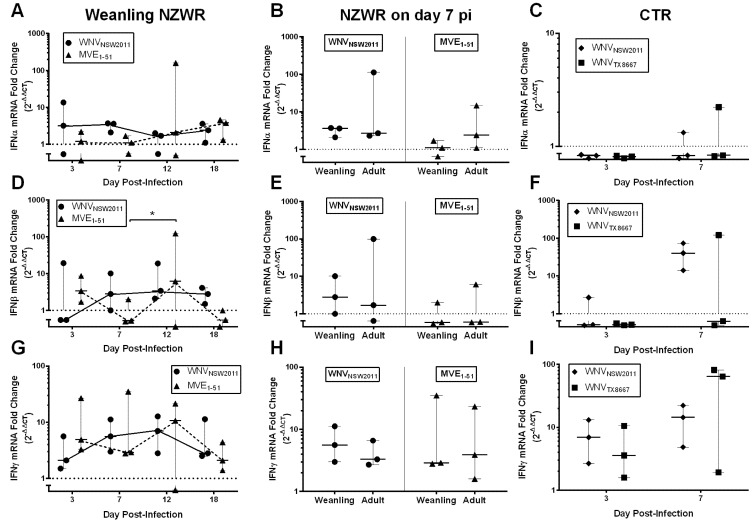
Type I and II IFN transcription kinetics in the draining PLN. IFNα (**A**–**C**), β (**D**–**F**) and γ (**G**–**I**) transcription in the draining politeal lymph node of weanling NZWRs (**A**,**D**,**G**) and CTRs (**C**,**F**,**I**). A comparison of the transcription profile on day 7 pi between weanling and adult NZWRs (**B**,**E**,**H**). Each data point represents the fold-change in transcription in one animal. Horizontal solid line in each time-point represents the median fold-change, with error bars indicating the range. Dotted line at Y = 1 signifies one-fold (basal transcription level). Two-way ANOVA with post-test Sidak was performed on the log_10_(Y+1) transformed mean of fold-change in each time-point. Statistical significance thresholds: * *p* ≤ 0.05, ** *p* ≤ 0.01, *** *p* ≤ 0.001, **** *p* ≤ 0.0001.

Overall, between WNV_NSW2011_ and MVE_1-51_ infected weanling NZWRs, there was a significant difference in the levels of IFN-I and -II transcription in the brain (Figure 4A,D,G; IFNα: F_1,16_ =18.6700, *p* = 0.0005; IFNβ: F_1,16_ = 9.377, *p* = 0.0074; IFNγ: F_1,16_ = 8.917, *p* = 0.0087), but not in the draining PLN (Figure 5A,D,G; IFNα: F_1,15_ = 0.0094, *p* = 0.9242; IFNβ: F_1,14_ = 0.1487, *p* = 0.7056; IFNγ: F_1,15_ = 0.9710, *p* = 0.3401).

IFNα, β, and γ transcription in the brain were comparable between the two groups on day 3 pi, but were quite different from day 7 pi onwards, with brains from MVE_1-51_-infected weanling NZWRs having consistently upregulated levels of IFNα, β, and γ transcripts (Figure 4A,D,G). In the draining PLN, an earlier upregulation of IFNα (from day 3 pi) and a more consistent upregulation of IFNβ (from day 7 pi) transcript levels were observed in WNV_NSW2011_-infected weanling NZWRs, when compared to the MVE_1-51_ group (Figure 5A,D). The IFNγ transcription kinetics in the draining PLN was similar for the two weanling NZWR groups (Figure 5G).

The levels of IFN-I and -II transcription in the brains of WNV_NSW2011_ and WNV_TX8667_-infected CTRs were similar to the profile seen in WNV_NSW2011_ infected weanling NZWRs, where only IFNγ mRNA was notably upregulated (Figure 4C,F,I). Upregulation of IFN-I transcription was only observed in brain of one CTR in the WNV_TX8667-_challenge group culled on day 7 pi (Figure 4C,F). For the draining PLN, infrequent upregulation of IFNα mRNA was detected on day 7 pi (Figure 5C, *n* = 1/3 for both CTR challenge groups). IFNβ transcription in the draining PLN was consistently upregulated in all three WNV_NSW2011_-infected CTRs culled on day 7 pi (Figure 5F). Only 1/3 of the WNV_TX8667_-infected rabbits on day 7 pi had elevated IFNβ transcription in the draining PLN (Figure 5F). The overall levels of IFNγ transcription in the draining PLN in the two CTR groups were comparable to that in the weanling NZWRs (Figure 5G,I). There was no significant effect of age on levels of IFN-I and -II transcription in the brain and draining PLN of NZWRs on day 7 pi, regardless of the virus strain inoculated (Figure 4 and Figure 5B,E,H; brain: IFNα: F_1,8_ = 0.1719, *p* = 0.6893; IFNβ: F_1,8_ = 0.5947, *p* = 0.4628; IFNγ: F_1,8_ = 2.215, *p* = 0.1750; draining PLN: IFNα: F_1,8_ = 1.839, *p* = 0.2121; IFNβ: F_1,8_ = 0.4166, *p* = 0.5367; IFNγ: F_1,8_ = 0.2250, *p* = 0.6479).

## 3. Discussion

The current study characterized the clinical, pathological, virological, and immunological outcomes in rabbits after peripheral WNV and MVEV infection. We observed a low-magnitude and transient viremia, similar to that seen in horses, NHPs, and humans [13,20,21]. Peripheral tissue tropism of WNV_NSW2011_ was restricted predominantly to the draining PLN, while MVE_1-51_ was more successful at disseminating to other lymphoid tissues, such as the contralateral PLN and spleen. However, regardless of the degree of peripheral dissemination, the inoculated virus was mostly undetectable in the brain. The associated early anti-WNV neutralizing antibody production in WNV-infected NZWRs and CTRs was also comparable to that in infected horses and humans [22,23,24]. This may be explained by the early IFNα/β and/or γ response in the draining PLN. Our study has also highlighted the importance of the innate immune response in early restriction of virus dissemination, before the appearance of neutralizing antibodies. The resultant mild clinical manifestation and neuropathology in infected rabbits are representative of the non-lethal disease reported for the majority of horse and human WNV infections [1,2,4,13]. Immunocompetent rabbits are, therefore, a useful animal model for investigating mechanisms of non-lethal neuropathogenesis and natural means of virus control against virulent WNV and MVEV challenge.

### 3.1. Experimental Design

In this study, we used NZWRs (*Oryctolagus cuniculus*) and CTRs (*Sylvilagus* sp.). By including CTRs in this study, we were able to investigate the effect of different rabbit genetic and potential immunological backgrounds on the outcomes of peripheral flavivirus challenge. We also employed the footpad intradermal inoculation route, commonly used in the mouse and hamster models (e.g., [7,8]). This allows appropriate comparison to natural human and equine mosquito bite inoculation. We chose an inoculation dose of 10^5^ TCID_50_ (for NZWRs) or PFU (for CTRs) per rabbit, as this is representative of a typical WNV dose delivered by a *Culex* sp. mosquito bite inoculation [25].

### 3.2. Rabbits Can Model Non-Lethal Human and Equine WNV Infections

**Figure 6:**
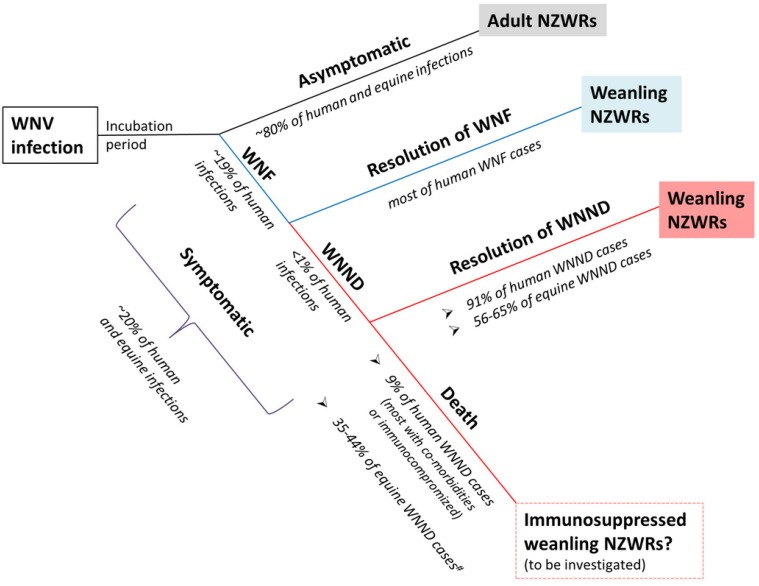
Schematic depiction of how NZWR can model the different aspects of human and equine WNV infections. Our study has shown that WNV-infected NZWRs produce a non-lethal phenotype that is representative of most human and equine infections. The asymptomatic nature of WNV infection in adult NZWRs makes this group of rabbits an appropriate model for determining factors responsible for asymptomatic resolution of human and equine WNV infections. Mild fever was observed in most weanling NZWRs infected with WNV, with some developing mild-moderate non-lethal neuropathology. This group is, therefore, a suitable model for investigating the mechanisms of WNV disease resolution. We also hypothesize that immunosuppression in weanling NZWRs may produce the lethal phenotype typically seen in fatal human and equine WNND cases. Further investigation is required for this particular aspect. This diagram used information obtained from [1,2,3,4,5,28]. ^#^ the reported equine case-fatality rate does not distinguish between euthanatized cases and natural deaths.

The WNV strain of main interest in this study was the Australian equine pathogenic strain, WNV_NSW2011_ [16]. WNV_NSW2011_ has previously been shown to be highly virulent in weanling and intermediately virulent in adult CD1 Swiss outbred mice, causing typical neurological signs, such as ataxia, tremor, and seizure prior to death [16]. We have shown in the current study that immunocompetent rabbits do not succumb to inoculation with this strain, regardless of age or species. In fact, even when inoculated with the highly murine-virulent MVE_1-51_ and a virulent North American WNV strain, WNV_TX8667_, no mortality was observed in either rabbit species. This contrasts with the moderate to high mortality often associated with MVE_1-51_ and North American lineage I WNV strains in the mouse and hamster model [7,8,26,27]. There were also no observable clinical signs, other than a mild to moderate febrile response on day 1 pi in weanling NZWRs. This relatively resistant phenotype in rabbits is representative of the majority of WNV infection in horses and humans [1,2,4,13]. Figure 6 summarizes how the NZWR model can be applied to study the different aspects of WNV infections in the humans and horses.

### 3.3. Productive Flavivirus Replication in Draining PLN

Both WNV and MVEV RNA were detected at substantial levels in the draining PLN of weanling NZWRs and CTRs. The detection of flavivirus NS1 antigen in these lymph nodes suggests that productive virus replication occurred in this tissue. Since the draining PLN is the only tissue where viral RNA and antigen were consistently detected across most groups, it may, therefore, be the main site of peripheral virus replication. We identified the permissive cells in these lymph nodes to be pleomorphic leukocytes, consistent with macrophages or dendritic cells in the paracortical zone (Figure 2C). Notably, scant NS1 positive leukocytes were also present in the dermis of a virus-inoculated footpad, suggesting that virions have been phagocytosed by leukocytes from the site of inoculation, as suggested in the mouse model [29,30]. Productive virus replication also occurred in these leukocytes, since flavivirus NS1 was expressed in these cells. But whether the virus was then transported inside phagocytes to the draining PLN remains to be determined.

There was a notable difference in the draining PLN viral load between the two age groups of NZWRs (Figure 3C). This indicates that adults may be more effective at controlling early peripheral virus replication than weanling NZWRs. This was further supported by the generally low viral burden in all tissues examined, the very mild neuropathology and the afebrile clinical course observed in adults. However, the levels of IFN-I and II transcription in the draining PLN and neutralizing antibody response were not significantly different between these two age groups (Figure 3I and Figure 5B,E,H). So the mechanisms for this very effective control of virus replication by adult NZWRs remain to be investigated. Characterization of the early immunological events prior to day 3 pi in infected adult and weanling NZWRs will likely shed light on the differences between the two age groups. Nevertheless, due to this benign phenotype, adult NZWRs may be a suitable model for investigating factors determining asymptomatic WNV infections in humans and horses (Figure 6).

### 3.4. Restricted Viremia and CNS Infection in Rabbits Resemble Human and Equine WNV Kinetics

Despite productive infection in the draining PLN, the viremia was low and transient for all virus challenge groups. Interestingly, the viremia peaks observed in our study were lower than those reported in previous flavivirus infection studies in rabbits (peak ~10^1−3^ TCID_50_ equivalent or PFU/mL, as compared to ~10^4−5^ CID_50_/mL or SMIC LD_50_/mL) [31,32]. This may be explained by the different experimental conditions involved, such as the use of different WNV strains [31], route of inoculation [32], trapping region for CTRs (e.g., Iowa [31]), virus isolation methods (CID_50_ for [31] and SMIC LD_50_ for [32]), and a potentially different adult to weaner ratio in recruited wild rabbit groups. Characterization of the effects of the mentioned methodological differences is beyond the scope of the current study, but will be an important aspect for future refinement of the rabbit model.

Nevertheless, the low viremia, restricted peripheral virus dissemination, and CNS infection in WNV-infected rabbits of this study resemble the *in vivo* virus kinetics reported for experimentally infected NHPs and horses [13,33]. Natural human and equine infections are also commonly associated with minimal to absent neural and extraneural infection [11,12,34]. We, therefore, suggest that the *in vivo* virus kinetics of WNV in immunocompetent rabbits is similar to that in healthy horses and humans.

### 3.5. Weanling NZWRs are an Appropriate Model to Study Non-Fatal Human and Equine WNND

Despite the lack of or only trace levels of viral RNA in the brain, mild to moderate non-lethal neuropathology was induced in flavivirus-infected rabbits, with animals infected with MVE_1-51_ and WNV_TX8667_ producing the most frequent and severe lesions (Figure 1A–C). The low case-fatality rate of 9% from WNND in humans suggests that non-lethal neurological disease is common (91%) [3]. The equine case-fatality rate of 35–44% also suggests that approximately half of the symptomatic horses survive WNV infection [5,28]. However, the true survival rate is likely to be higher, since the reported equine case-fatality rate does not distinguish between euthanized cases and natural deaths [5]. This highlights the relatively common occurrence of WNV-induced non-lethal neurological disease in the incidental hosts, yet the mechanism of which remains poorly understood. Furthermore, the economic impact of non-lethal WNV-induced disease in human is substantial, highlighting the significance of investigating this important presentation of the disease [6]. The natural development of mild-moderate non-lethal neuropathology mainly in flavivirus-challenged weanling NZWRs suggests that this group of rabbits may be an appropriate model to study the mechanisms of non-lethal neuropathogenesis in horses and humans (Figure 6). Further investigation of the expression kinetics of pro- and anti-inflammatory cytokines in neural and extraneural tissues may provide insights into the mechanisms behind the induction and resolution of neuropathology in flavivirus-infected weanling NZWRs.

### 3.6. Virus-Dependent Systemic Virus Dissemination, Peripheral IFN-α/β, and Neutralizing Antibody Response

The degree of systemic virus dissemination was virus-dependent in weanling NZWRs. MVE_1-51_ appeared to be more successful in establishing infection in the non-draining PLN and spleen, when compared to WNV_NSW2011_ (Figure 3F). This was not surprising given the higher virulence of MVEV than WNV_NSW2011_ in mice [16,35]. The slower induction of IFNα response in the draining PLN (Figure 5A) and neutralizing antibody production (Figure 3G) in MVE_1-51_-infected weanling NZWRs, relative to ones infected with WNV_NSW2011_, likely have contributed to the more successful peripheral virus dissemination. This association between a delayed peripheral immune response and more profound peripheral virus replication has been reported in mice infected with highly virulent WNV strains, such as WNV_KOU_ [9].

However, contrary to the mouse model, weanling NZWRs infected with MVE_1-51_ did not establish widespread CNS infection. There were trace levels of MVE_1-51_ RNA in the brain of some of the infected weanling NZWRs. But the elevated and sustained brain IFN-I/II response in MVE_1-51_-infected weanling NZWRs likely played an important role in local virus control, especially when a delayed peripheral immune response failed to effectively restrict early viral dissemination (Figure 4A,D,G).

Conversely, the faster and more robust IFNα/β response in the draining PLN against WNV_NSW2011_ in weanling NZWRs may explain the limited dissemination of virus to the non-draining PLN and spleen (Figure 3F). Direct IFNα/β restriction on WNV replication, especially for less pathogenic WNV strains, has been well documented [36,37,38]. However, this faster induction of IFN-I in the draining PLN may also be important for the development of a rapid and effective neutralizing antibody response [39]. In fact, the kinetics of neutralizing antibody production in WNV-infected rabbits mimics very closely that of WNV-infected horses, where PRNT_90_ neutralizing antibody titers of 10 to 80 were reported on day 7 pi [22]. Castillo-Olivares *et al.* also detected anti-WNV neutralizing antibody (~100% neutralization at 1:10 serum dilution) from day 8 pi in experimentally-infected horses [23]. This similarity may also apply to humans, since IgM seroconversion in humans has been estimated to occur around day 7 to 8 post-WNV infection (~4 days after peak viremia) [24,40]. This early neutralizing antibody production in WNV_NSW2011_ infected weanling NZWRs may have provided adequate peripheral protection against viral neuroinvasion around the crucial time of day 6 to 7 pi, when flavivirus neuroinvasion typically occurs in mice [7]. This may explain the absence of WNV_NSW2011_ RNA in the brain of these animals. The lack of the continual stimulus in the brain, in turn, corresponds to the lack of upregulated brain IFNα/β transcription in these animals. The contrary was observed in MVE_1-51_ infected weanling NZWRs, in which the slower neutralizing antibody production may have allowed viral neuroinvasion, which in turn induced a higher and more sustained upregulation of brain IFNα/β transcripts (Figure 4A,D).

While we have highlighted the delayed neutralizing antibody production in MVEV-infected NZWRs as a potential reason for the more extensive virus dissemination in the periphery, the previous rabbit study by Kay *et al.,* reported an earlier detection of anti-MVEV neutralizing antibodies (titer of ~<10–20) in MVEV-infected feral Australian rabbits (*Oryctolagus cuniculus*) on day 7 pi [32]. However, these feral rabbits may have had a different immunological background to laboratory NZWRs, since co-infection with viruses, bacteria, fungi and/or parasites may have pre-stimulated the immune system in feral rabbits.

### 3.7. Neutralizing Antibody and IFNγ Response in CTRs

Evidence of higher neutralizing antibody titers in feral rabbits (CTRs), as compared to laboratory NZWRs, was also observed in the current study (Figure 3H). Since the level of neutralizing antibody production has been reported to be associated with the intensity of IFNγ induction in lymph nodes [41], the higher neutralizing antibody production in CTRs may be explained by the marginally higher IFNγ transcription in the draining PLNs of the CTRs compared to the NZWRs especially on day 7 pi (Figure 5G,H,I). Furthermore, upregulated IFNγ expression in the spleen and the CNS has been reported to be associated with peripheral parasitic infection in mice [42,43]. Potential background parasitic infection in feral CTRs may, therefore, play an important role in shaping the IFNγ and the associated neutralizing antibody response in flavivirus-challenged feral rabbits. Wild rabbits may, therefore, be a useful model for investigating the interactions between parasitic and flavivirus co-infection.

### 3.8. IFNγ Response in NZWRs

Early upregulated IFNγ transcription was also observed in the draining PLN and the brain of most flavivirus-challenged NZWRs (Figure 4 and Figure 5G,H,I). The only exception was in the brain of adult NZWRs infected with WNV_NSW2011_. This upregulation in IFNγ transcripts may have provided early peripheral virus control before a detectable level of neutralizing antibodies began to circulate (prior to day 7 pi for WNV-infected rabbits and prior to day 12 pi for MVE_1-51_ infected rabbits). The suggested mechanism of virus control by IFNγ is via its activation of macrophages/dendritic cells/microglial cells [44,45], its support role in establishing a robust CD8+ T cell response [46], and its role in shaping the antibody response by class switching from IgM to IgG virus neutralizing antibodies [47]. IFNγ also has an enhancement role in IFNα/β induction via the activation of interferon responsive factor I (IRF-1) [48]. We propose that IFNγ may play a key role in: (i) the control of viral load in the draining PLN by its activation of infected macrophages/dendritic cells; (ii) controlling virus infection in the brain by its activation of microglial cells and/or the infiltrated T cells; (iii) forming a competent neutralizing antibody response by class switch; and (iv) co-activation of the IFN-I induction in the draining PLN and brain.

Activated T lymphocytes, γδ T lymphocytes and natural killer (NK) cells have been reported to be the main producers of IFNγ [49,50,51]. It was, therefore, not surprising to observe IFNγ transcript upregulation in brains with evidence of CD3+ T cell infiltration (Figure 2A,B). In fact, CD3+ T cells infiltration into the CNS was a common observation in human, NHP, and equine WNV infections [12,52,53]. Furthermore, upregulated IFNγ gene transcription in nucleated cells isolated from CSF samples of WNV-infected horses has been reported [54]. This highlights the importance of T lymphocytes and likely the associated IFNγ response in virus control in the CNS in incidental hosts and rabbits.

Notably, upregulated IFNγ transcription could also be observed in rabbit brains without pathology. While it is possible that rare CNS-infiltrating NK or T cells were not within the histological section examined, a non-leukocyte source(s) of IFNγ may be possible. In aged BALB/c mice, expression of IFNγ and its receptor has been reported in the cerebral and cerebellar microvascular endothelium, as well as in the choroid plexus [55]. While this did not apply to young adult mice, characterization of such age-related differences has not been performed in rabbits. Furthermore, expression of IFNγ and its receptor has been reported in rat sensory neurons grown in culture [56]. These potential non-leukocytic sources of IFNγ should be investigated further in our rabbit model by IHC or *in situ* hybridization for mRNA.

The large variation, as indicated by the large error bars, observed in the rabbits’ response against WNV and MVEV infection is likely explained by their outbred background. However, despite this large variation, all rabbits consistently controlled virus replication and survived virus challenge without display of severe clinical signs. This suggests that virus control in outbred rabbits can be achieved at various efficiencies. It is likely that such variability exists in natural human and equine WNV infections, as suggested by the large range of disease outcomes (Figure 6). The outbred rabbit model may, therefore, be a more appropriate tool for delineating the reasons for such variability than the typical inbred mouse models.

### 3.9. Significance and Future Directions

Little is known regarding how virulent flaviviruses induce non-lethal disease and are controlled in naturally resistant hosts. We have presented an alternative small animal model in rabbits for studying WNV-induced non-lethal neuropathogenesis and the natural mechanisms of virus control. We have demonstrated that rabbits, regardless of age or species, are consistently resistant to virulent WNV challenge. As such, rabbits are more suited for investigating mechanisms of virus control than the relatively more susceptible mouse model. Furthermore, this highly resistant phenotype is representative of most human and equine WNV infections. Better understanding of non-lethal neuropathogenesis and resolution of virus infection will help identify key molecules relevant for improving antiviral and therapeutics design for reducing WNV-associated morbidity and mortality in humans and horses. Identification of the factors that determine survival will also help identify novel prognostic indicators for disease progression. As further outbreaks of WNV are expected across the world, non-lethal WNV disease will continue to have great economic impact on global healthcare systems [6,57]. The establishment of the rabbit model presented in this study is, therefore, an important tool for devising ways of ameliorating the impact of WNV on global health.

## 4. Materials and Methods

### 4.1. Cell Culture and Virus

African green monkey (Vero) and *Aedes albopictus* salivary gland (C6/36) cells were cultured as previously described [9]. Three strains of flaviviruses were used in this study. The isolation, propagation and characterization of the equine pathogenic WNV_NSW2011_ outbreak strain and the prototype MVE_1-51_ have previously been described in details [16,26,58]. An additional passage on C6/36 cells was performed prior to use for inoculation. The North American WNV_TX8667_ was kindly provided by Robert Tesh, University of Texas Medical Branch (Galveston, TX, USA) and was originally isolated from a bluejay during the 2012 human outbreak in Texas; the virus was passaged once in Vero cells prior to use.

### 4.2. Animals and Experimental Design

Two species of rabbits, the laboratory New Zealand White (NZWR; *Oryctolagus cuniculus*) and the North American cottontail rabbit (CTR; *Sylvilagus* sp.), were used in this study. Experimental infection of these two species of rabbits was conducted separately in PC2 and BSL3 animal holding facilities at University of Queensland (UQ), Australia, and Colorado State University (CSU), USA, respectively. Ethical approval was granted from the UQ Animal Ethics Committee (SVS/369/12/ARC) and the CSU Institutional Animal Care and Use Committee (#14-5170). In total, 57 NZWRs were sourced from Nanowie Small Animal Production Unit (Belbrae, Victoria), while 14 CTRs were trapped in peri-urban areas of Fort Collins, CO, USA. All rabbits used in this study were seronegative by a plaque reduction neutralization test to WNV and MVEV prior to the experimental infection.

The study was divided into three parts: a pilot experiment to explore an age-dependent susceptibility of NZWRs to flavivirus infection, a large scale study to characterize the kinetics of virus infection and pathology, and thirdly, a comparative kinetics study in CTRs. Assignment of rabbits into each virus challenge group is outlined in Table 1. Additionally, six NZWR and two CTR controls were sham-inoculated with cell culture media.

For all experiments, a dose of 10^5^ TCID_50_ (for NZWRs) or PFU (for CTRs) in 50 µL of cell culture media was inoculated intradermally in the left hind footpad. Inocula used for the infections were back-titrated and confirmed to be within one log_10_ of the expected amount. Scheduled euthanasia was performed by overdose with pentobarbitone (Lethabarb, Virbac Animal Health) injection and terminal cardiac bleed.

### 4.3. Clinical Monitoring

A registered veterinarian performed daily clinical monitoring of infected rabbits for signs of malaise and neurological deficit. Measurements of rectal (for NZWRs) or body (for CTRs) temperature, as well as their weights were taken daily. For NZWR rabbits, a standard electronic thermometer was used per rectum. An IPTT300 temperature transponder (BioMedic Data Systems, Inc., Seaford, DE, USA) was implanted subcutaneously in CTRs for daily temperature measurement. The approximate size of both the draining and contralateral PLNs was also gauged by palpation in the NZWR groups. The general alertness, as well as food and water intake, were used as indicators of their well-being. Daily neurological examination by the hopping test was performed on NZWRs to observe for significant loss of coordination of hind and forelimbs [59]. Other neurological signs such as seizures and muscle twitching were monitored.

### 4.4. Tissue Sampling

From day 1 to 7 pi, blood was collected daily in EDTA-coated or plain collection tubes from the ear vein. Additionally, terminal cardiac bleeds were performed at the time of euthanasia. Collected blood was centrifuged. Plasma/serum was extracted and immediately frozen at −80 °C for virus isolation, viral RNA quantitation, and antibody assays.

Following euthanasia, a complete post-mortem examination was performed. Both neural (olfactory bulbs, brain, spinal cord, eyes and sciatic nerves) and extraneural (PLNs, spleen, tracheobronchial lymph nodes, adrenals, kidneys, liver, lungs, heart and thymus) tissues were harvested for one to three of the following assays: histopathological analysis, virus isolation, and viral RNA quantitation.

### 4.5. Histopathology

Samples were fixed in 10% neutral buffered formalin solution for 48 to 72 h. They were then transferred to 70% ethanol for storage until routine paraffin embedding was performed. Five micrometer sections were stained with hematoxylin and eosin (H&E) and examined on a Nikon YS100 light microscrope. Microphotographs were produced as previously described [9]. Histopathological scoring was performed independently by two pathologists, one of whom was blinded to the groups. Specific for the neural tissue, multiple transverse sections of the brain (5–6) and spinal cord (9–10) were examined. The olfactory bulbs and pituitary glands were analyzed and described separately from the rest of the brain. Scores were assigned according to the severity and extensiveness of the lesions. The data presented are the consensus scores between the two pathologists. For neural tissues, the score matrix outline in Table 4 was used.

**Table 4:** Neuropathology score matrix.

**Extensiveness**		**SEVERITY**		
		**Mild**	**Moderate**	**Severe**
	Focal	1	2	3
	Multifocal	2	3	4
	Diffuse	3	4	5

For lymphoid tissues, such as lymph nodes and spleen, a three-tiered scoring system was used to gauge the level of immunological activation, with a score of 3 indicating the most pronounced activation.

### 4.6. Immunohistochemistry (IHC)

Immunolabeling for flaviviral NS1 antigen has been previously described in detail [9]. Immunophenotyping of rabbit T lymphocytes and histiocytes in inflammatory aggregates was achieved using cross-reactive anti-human CD3 (CD3 clone F7.2.38, Dako) and anti-human myeloid/histiocytic antigen (Clone MAC387, Dako) mouse monoclonal antibodies, respectively, using previously described protocols [60]. For each IHC batch, a positive and negative antigen control was included.

### 4.7. Virus Isolation and Titration

For NZWR samples, virus isolation and titration was determined by 50% endpoint of tissue culture infective dose (TCID_50_) [9]. Ten-fold serial dilution in 2% FBS-DMEM of sera or 10% w/v tissue homogenates was performed [9]. Preparation of homogenates has been previously described [9]. Fifty µL of each dilution was added to a subconfluent monolayer of Vero cells in each well of a 96-well tissue culture plate (Costar, Corning). Six to ten replicate wells were used per dilution. The plates were incubated in a 37 °C incubator at 5% CO_2_ for 5 days, before fixation with 20% acetone (supplemented with 0.02% BSA) for 12–24 h at 4 °C. Immunocytochemistry was performed on fixed cells, which involved blocking buffer incubated for one hour, anti-flavivirus E protein monoclonal antibody, 4G2, incubated for one hour [61], and HRP-conjugated goat-anti-mouse antibody incubated for 30 minutes, all at room temperature. Staining of 4G2 positive Vero cells in each well was achieved using DAB+ substrate (Dako) at 1:50 dilution. Each well was manually examined for positive cells under an inverted light microscope (Olympus LH50A). For each assay, both positive and negative controls confirmed the validity of the results.

For CTR samples, standard plaque assay was performed on ten-fold dilutions of sera and 10% weight/volume tissue homogenates, previously described in [13,62]. Hundred µL of inocula was added to confluent monolayers of Vero cells in 6-well tissue culture plates (Costar, Corning) and incubated for 1–2 h at 37 °C, before 2 mL of 0.5% agarose gel overlay was added to each well. Plates were incubated at 37 °C 5% CO_2_ for 2 days before 2 mL of a second gel overlay containing 0.005% neutral red stain was added to each well. Plaques were read on the third and fourth day pi. In each run, both positive and negative controls confirmed the validity of the results.

### 4.8. Serology

An initial screen for flavivirus specific antibodies was performed on plasma/sera obtained from NZWR rabbits at termination by a 4G2 pan-flavivirus blocking ELISA [63,64].

Specific anti-WNV_NSW2011_ and MVE_1-51_ neutralizing antibodies in plasma/sera were assayed for by plaque reduction neutralization test (PRNT_90_) in Vero cell culture, as previously described in [13,62]. Plasma/serum concentrations tested were 1:10 to 1:320, diluted in culture media (DMEM). For screening exposure status of pre-inoculated rabbits and control rabbits, only a 1:10 dilution of the plasma/serum was tested. Plasma/serum dilutions were then incubated with equal volume (100 uL) of diluted virus stock for 1 at 37 °C. Hundred µL of the plasma/serum-virus mixture was used to inoculate subconfluent monolayer of Veros cells in each well of a 6-well tissue culture plate (Costar, Corning), as per plaque assay protocol [13,62], with modification of agarose used (1% of SeaPlaque, Lonza) and method of plaque visualization (1:40 dilution of neutral red dye (Sigma Aldrich) in sterile PBS, applied over the first gel overlay on day 3 pi). Plaques were counted on the fourth day after inoculation. Concentration of WNV_NSW2011_ and MVE_1-51_ inocula was back-titrated at neat, 1:2 and 1:4 dilutions in duplicate wells along with each assay to determine the number of plaque forming units used to incubate with plasma/serum dilutions. Neutralizing antibody titers were determined as the inverse of the last plasma/serum concentration showing 90% plaque reduction.

### 4.9. RNA Extraction

Viral/total RNA was extracted from plasma/sera and tissues, using Qiamp viral RNA (Qiagen) and RNeasy lipid tissue RNA extraction kit (Qiagen), respectively. Protocol as outlined by the kit was followed, except for tissue harvested from CTR rabbits. For these samples, Qiazol (Qiagen) was substituted with Trizol (Invitrogen). Tissue was homogenized in 1 mL of Qiazol or Trizol using an Omni-tip homogenizer (Omni) or stainless steel beads with a mixer mill (Retsch, Inc., Newtown, PA, USA), respectively. The remaining steps were performed according to the manufacturer’s protocol. Additional DNase I digest in solution was performed on extracted RNA, followed by additional RNA clean up with RNeasy RNA extraction kit (Qiagen). The absence of genomic DNA was confirmed by performing PCR without reverse-transcription on a selection of RNA samples from each extraction batch. Quality and concentration of extracted RNA was determined by Nanodrop 1000 spectrophotometer (Thermo Scientific). RNA samples were kept at −80 °C.

### 4.10. Viral RNA Quantitation (qRT-PCR)

Quantitative reverse-transcriptase PCR (qRT-PCR) was used for viral RNA quantitation. Two µL of RNA template was added in an optimized 15 µL reaction, using the one-step Rotor Gene SYBR green RT-PCR kit (Qiagen). Primers targeting WNV_KUN_ and MVE_1-51_ specific NS5 [65] were used at 0.25 µM of the final PCR reaction volume. Concentrations of the remaining reagents were used as recommended by the kit. Rotor gene 6000 platform (Qiagen) was used for qRT-PCR amplification. Optimized cycling conditions for the WNV_NSW2011_ assay involved reverse transcription at 55 °C for 10 min, then PCR initial activation at 95 °C for 5 min, followed by 45 cycles of 95 °C for 5 s, 65 °C for 10 s and 72 °C for 5 s. The MVE_1-51_ assay cycling conditions were identical to that of WNV_NSW2011_, except the annealing temperature was 60 °C [65]. Standard melt curve analysis was performed at the end of each run to check for appropriate qRT-PCR amplification. Standard curve was also generated for both WNV and MVEV assays by serial dilutions of extracted RNA from previously titrated virus stock, similar to method described by Lanciotti *et al.* [66]. Minimal difference was detected when standard curve was generated from RNA extracted from previously diluted virus stock in control plasma.

In each run, three biological replicates of plasma/serum or tissue sample per time-point, no template control, uninfected tissue controls, and viral RNA positive controls were included. The viral RNA titer in plasma/sera was expressed as TCID_50_ equivalent/mL. For tissue viral load, normalization of viral RNA CT was performed first against the housekeeping gene glyceraldehyde 3-phosphate dehydrogenase (GAPDH), primers for which have been published in [67]. The 2^−^^ΔΔCT^ method was used to quantify tissue viral load relative to background fluorescence from sham-infected controls [68,69]. This relative viral burden was expressed as fold increase over sham-infected controls. Limit of detection of both the WNV and MVEV assays was around 29 and 6 TCID_50_ equivalent/mL, as determined by limiting serial 10-fold dilutions.

### 4.11. Cytokine Transcription Profiling Assay (RT-qPCR)

IFNα, β, and γ transcription profiling was performed by two-step RT-qPCR. Two µL of RNA was used in a 20 µL qScript first strand synthesis reaction (Quanta Bioscience), which consists of 4 µL of qScript reaction mix (5×) containing a mix of oligo-dT and random primers, and 1 µL of qScript reverse transcriptase. The remaining volume was made up by nuclease free water. First strand synthesis reaction was performed on Eppendorf mastercycler epGradient S PCR thermal cycler (Eppendorf), with cycling conditions involving 5 min of 22 °C for 5 min, 42 °C for 30 min and 85 °C for 5 min. Two µL of cDNA was used in a 20 µL Rotor Gene SYBR green PCR reaction (Qiagen); 0.25 µM of each forward and reverse primer was used for each reaction. Concentration of the remaining reagents was used as recommended by kit’s protocol. IFNβ and γ primers have been previously described in [67]. Primers for IFNα were designed from NCBI reference sequence XM_002708065.1 (IFNα-5-like mRNA) using primer design software, Primer 3 plus. IFNα forward primer sequence is 5'-TGCTTGCAGGACAGACATGA-3' and reverse primer sequence is 5'-ATCTCTGTGCTCCACGAGAT-3'. Validation of this primer pair was performed with gel electrophoresis of qPCR products to confirm the 95 base-pair long amplicons. Rotor Gene 6000 platform was used for qPCR amplification with a common cycling conditions for all primer sets, which involves a PCR initial activation step of 95 °C for 5 min, followed by 40 cycles of 95 °C for 5 s, 60 °C for 10 s and 72 °C for 5 s. Standard melt curve analysis was performed at the end of each run to confirm appropriate PCR amplification. In each run, one cytokine mRNA positive control, generated from concanavalin A (Sigma Aldrich) challenged *ex vivo* rabbit splenocyte culture, was used. No template control, as well as control tissue samples from sham-inoculated rabbits, was included in each run. Cytokine transcription in sample was normalized to that of the house-keeping gene GAPDH (Δ CT_1_) [67], and then calibrated to the normalized basal transcription in sham-inoculated control animals (Δ CT_2_). The 2^−^^ΔΔCT^ method was then used to calculate the relative fold change in transcription of each sample over that of the controls [68].

### 4.12. Statistical Analysis

Clinical parameters, such as rectal temperature, were normally distributed. Virus titers from various tissues and sera/plasma, as well as antibody titers and cytokine transcription fold-change, were log_10_(Y+1) transformed in order to establish normal distribution. A two-way ANOVA with post-hoc multiple comparisons was performed on these data, in order to assess the effect of time, inoculated virus strain, age, and species of rabbits on the measurements mentioned above. *p*-values have been adjusted for multiple comparisons by either the Tukey or the Sidak method. One-way ANOVA or unpaired t-test with Welch’s correction was performed as appropriate. Fisher’s exact test was used to compare proportions of animals in each group with pathology. All statistical analyses were performed in statistical software, Graphpad Prism 6.

## 5. Conclusions

The use of the mouse and hamster model for studying host control of virulent WNV infections is limited by their propensity to developing lethal CNS infection. Furthermore, most human and equine WNV infections are not fatal, highlighting the incompatibility of rodents to model the pathogenesis in the incidental hosts. The resistant phenotype induced in WNV-infected rabbits, observed in the current study, is representative of the non-lethal presentation of most human and equine infections. This suggests that rabbits are an appropriate alternative model for studying the induction and resolution of WNV-associated disease. As further outbreaks of WNV are anticipated globally, better understanding of the mechanisms of virus control is required, in order to identify novel prognostic indicators, and improve therapeutic and preventative strategies. The current study has, therefore, laid the foundation for addressing this important gap in knowledge.

## Supplementary Information

Supplementary information can be accessed at: http://www.mdpi.com/2076-0817/4/3/529/s1.
