# Supplementary material for: Experimental West Nile Virus Infection in Rabbits: An Alternative Model for Studying Induction of Disease and Virus Control
Source: Pathogens. 2015 Jul 14;4(3):529–58. doi: 10.3390/pathogens4030529 (PMC4584271; doi:10.3390/pathogens4030529)
Supplement: Supplementary file 1 [file pathogens-04-00529-s001.pdf]

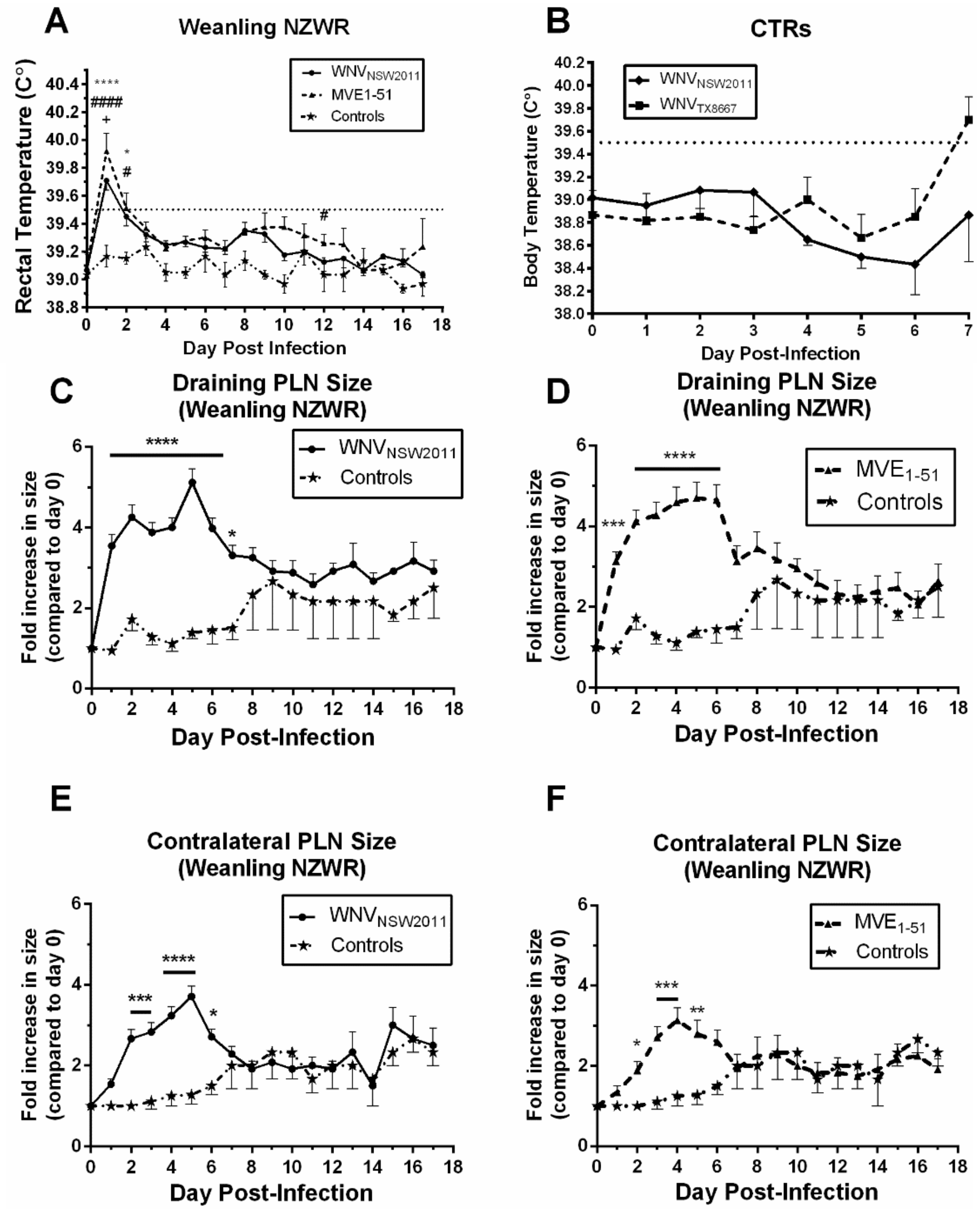

Figure S1. Clinical parameters in weanling NZWRs and CTRs. Daily rectal temperature in NZWRs (**A**) and body temperature in CTRs (**B**). Dotted line at 39.5 °C indicates the normal upper limit of rectal/body temperature. Fold-increase in the size of the popliteal lymph nodes over the initial size on day 0 before inoculation (**C**–**F**). Each point and error bar on the line indicate the mean and SEM respectively. *n* = 21–24 for days 1–3 pi, 18–21 for days 4–7 pi, 12 for days 8–12 pi, and 6 for days 13–17 pi. Two-way ANOVA with post-test Tukey was performed on the means of the measurements in each time-point. For panel A, comparison between the $WNV_{NSW2011}$ group and controls (*), between the $MVE_{1\text{-}51}$ group and controls (#), and between the $WNV_{NSW2011}$ and $MVE_{1\text{-}51}$ groups (+). For panels C-F, statistical analysis was performed on the mean fold change of virus challenge groups to that of the control group (*). Statistical significance thresholds: * or # or + $p \leq 0.05$, ** or ## or ++ $p \leq 0.01$, *** or ### or +++ $p \leq 0.001$, **** or #### or ++++ $p \leq 0.0001$

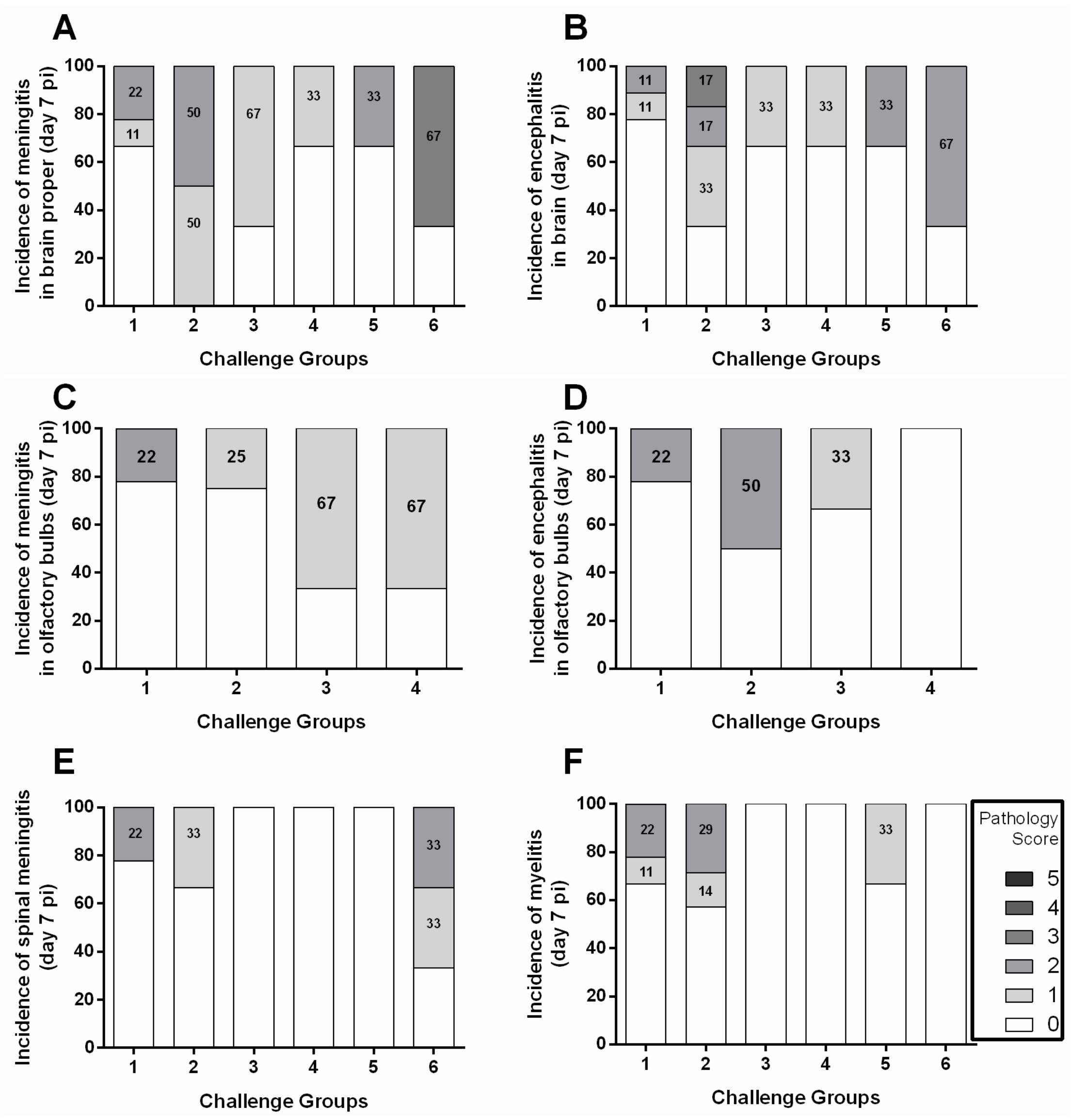

Figure S2. Incidence and severity of neuropathology on day 7 pi amongst the different virus challenge groups. Incidence of meningitis and encephalitis in the brain (**A**,**B**), meningitis and encephalitis in the olfactory bulbs (**C**,**D**), and spinal meningitis (**E**) and myelitis (**F**). Severity of neuropathology is indicated by the shade of the bar slices (darkest indicates most severe). Number in each bar slice indicates the percentage that the slice represents. Group 1: $WNV_{NSW2011}$ infected weanling NZWRs (n = 9); Group 2: MVE_{1-51} infected weanling NZWRs (n = 6); Group 3: $WNV_{NSW2011}$ infected adult NZWRs (n = 3); Group 4: MVE_{1-51} infected adult NZWRs (n = 3); Group 5: $WNV_{NSW2011}$ infected CTRs (n = 3); Group 6: WNV_{TX8667} infected CTRs (n = 3).

Table S1. Frequency of flavivirus NS1 positive leukocytes and lymphocyte activation in the draining popliteal lymph node

	**Day 3 pi**		**Day 7 pi**	
	**IHC (NS1)**	**Activation**	**IHC (NS1)**	**Activation**
$WNV_{NSW2011}$ (weanling NZWR)	2/2	1/2	0/2	5/5
MVE_{1-51} (weanling NZWR)	2/2	2/2	0/2	4/4
$WNV_{NSW2011}$ (CTR)	2/2	2/3	1/2	2/2
WNV_{TX8667} (CTR)	1/2	1/2	1/2	3/3

Table S2. Number of rabbits needed to infect (number needed to harm) before one animal is expected to have the following neuropathology on day 7 pi. The higher number needed to harm (NNH) associated with $WNV_{NSW2011}$ infected weanling NZWRs (Group 1), as compared to ones infected with MVE_{1-51} (Group 2), suggests that $WNV_{NSW2011}$ is less consistent in causing neuropathology than MVE_{1-51} in weanling NZWRs. This may be an indicator of the relatively lower pathogenicity of the $WNV_{NSW2011}$ strain.

Groups	Meningitis (Brain)	Encephalitis (Brain)	Meningitis (Olfactory Bulbs)	Encephaliitis (Olfactory Bulbs)	Spinal Meningitis	Myelitis
1	3	5 (4.5)	5 (4.5)	5 (4.5)	5 (4.5)	3
2	1	2 (1.5)	4	2	3	3 (2.3)
3	2 (1.5)	3	2 (1.5)	3	NA	NA
4	3	3	2 (1.5)	NA	NA	NA
5	3	3	ND	ND	NA	3
6	2 (1.5)	2 (1.5)	ND	ND	2 (1.5)	NA

Number in brackets is the raw results before the figure was rounded to the nearest integer. NA, unable to calculate NNH, since there was no reported incidence of that particular pathology. ND, not done, since these sites were not examined. Group 1: $WNV_{NSW2011}$ infected weanling NZWRs ($N = 9$); Group 2: MVE_{1-51} infected weanling NZWRs ($N = 6$); Group 3: $WNV_{NSW2011}$ infected adult NZWRs ($N = 3$); Group 4: MVE_{1-51} infected adult NZWRs ($N = 3$); Group 5: $WNV_{NSW2011}$ infected CTRs ($N = 3$); Group 6: WNV_{TX8667} infected CTRs ($N = 3$).

Table S3. Incidence of neuritis and peri-/epi-neuritis in sciatic nerves.

	Day 3 pi		Day 7 pi	
	Ipsilateral Sciatic Nerve	Contralateral Sciatic Nerve	Ipsilateral Sciatic Nerve	Contralateral Sciatic Nerve
$WNV_{NSW2011}$ (weanling NZWR)	2/2	ND	0/5	0/3
MVE_{1-51} (weanling NZWR)	3/3	1/3	1/4	0/3
$WNV_{NSW2011}$ (adult NZWR)	ND	ND	0/3	0/3
MVE_{1-51} (adult NZWR)	ND	ND	0/3	1/3
$WNV_{NSW2011}$ (CTRs)	1/3[#]	1/3	0/3	2/3
WNV_{TX8667} (CTRs)	0/3	0/3	0/3	2/3

[#] neuritis, N/D: not done.

© 2015 by the authors; licensee MDPI, Basel, Switzerland. This article is an open access article distributed under the terms and conditions of the Creative Commons Attribution license (http://creativecommons.org/licenses/by/4.0/).
